# The Spicy Story of Cannabimimetic Indoles

**DOI:** 10.3390/molecules26206190

**Published:** 2021-10-14

**Authors:** Allyn C. Howlett, Brian F. Thomas, John W. Huffman

**Affiliations:** 1 Department of Physiology and Pharmacology, Wake Forest School of Medicine, Winston-Salem, NC 27157, USA; 2 Department of Analytical Sciences, The Cronos Group, Toronto, ON M5V 2H1, Canada; brian.f.thomas01@gmail.com; 3 Department of Chemistry, Clemson University, Clemson, SC 29634, USA; huffman@clemson.edu

**Keywords:** aminoalkylindole, allodynia, antinociception, cannabinoid receptor, CP55940, JWH-018, K2, pravadoline, spice, WIN55212-2

## Abstract

The Sterling Research Group identified pravadoline as an aminoalkylindole (AAI) non-steroidal anti-inflammatory pain reliever. As drug design progressed, the ability of AAI analogs to block prostaglandin synthesis diminished, and antinociceptive activity was found to result from action at the CB_1_ cannabinoid receptor, a G-protein-coupled receptor (GPCR) abundant in the brain. Several laboratories applied computational chemistry methods to ultimately conclude that AAI and cannabinoid ligands could overlap within a common binding pocket but that WIN55212-2 primarily utilized steric interactions via aromatic stacking, whereas cannabinoid ligands required some electrostatic interactions, particularly involving the CB_1_ helix-3 lysine. The Huffman laboratory identified strategies to establish CB_2_ receptor selectivity among cannabimimetic indoles to avoid their CB_1_-related adverse effects, thereby stimulating preclinical studies to explore their use as anti-hyperalgesic and anti-allodynic pharmacotherapies. Some AAI analogs activate novel GPCRs referred to as “Alkyl Indole” receptors, and some AAI analogs act at the colchicine-binding site on microtubules. The AAI compounds having the greatest potency to interact with the CB_1_ receptor have found their way into the market as “Spice” or “K2”. The sale of these alleged “herbal products” evades FDA consumer protections for proper labeling and safety as a medicine, as well as DEA scheduling as compounds having no currently accepted medical use and a high potential for abuse. The distribution to the public of potent alkyl indole synthetic cannabimimetic chemicals without regard for consumer safety contrasts with the adherence to regulatory requirements for demonstration of safety that are routinely observed by ethical pharmaceutical companies that market medicines.

## 1. Introduction: Pravadoline and the Discovery of Aminoalkylindole Analgesics

**Figure 1:**
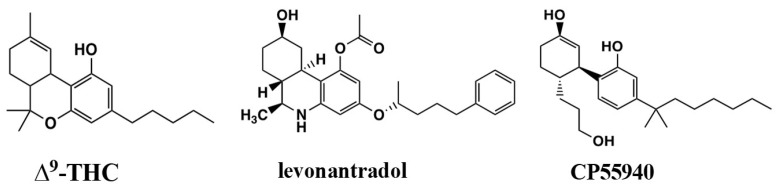
Classical cannabinoids Δ^9^-tetrahydrocannabinol (THC) and levonantradol and non-classical A,C-bicyclic cannabinoid CP55940.

The Howlett laboratory entered the cannabinoid field from the investigation of analgesic compounds that chemists at Pfizer Central Research had developed [1,2,3] in their quest to introduce a non-opioid, non-aspirin-like analgesic based upon the structure of the active 11-hydroxylated metabolite of Δ^9^-tetrahydrocannabinol (THC) [4,5] (Figure 1). Pfizer discontinued the cannabinoid analgesic program after early clinical trials with levonantradol (Figure 1) [5,6,7,8,9] but left a legacy of promoting cannabinoid therapeutics within the scientific research community (see symposium covering chemistry, biochemistry, pharmacokinetics, pharmacotherapeutic uses, government regulations, and philosophical considerations [10]). Investigations in the Howlett laboratory identified that the antinociceptive activity of the classical and nonclassical cannabinoid ligands was associated with their agonist activity at a G-protein-coupled receptor (GPCR) coupled to Gi that could inhibit cAMP accumulation [1,2,11,12,13]. These studies led to the development of a radioligand binding assay using [^3^H]CP55940 (Figure 1) to characterize the cannabinoid receptor in neuronal cells and the brain [12,14,15,16]. As these studies were being published, Dr. Howlett was contacted by Dr. Susan Ward at Sterling Research Group of Sterling Winthrop, Inc. (a subsidiary of Eastman Kodak), inquiring about whether the Howlett lab would be able to screen analgesic compounds that did not fit the pattern for non-steroidal anti-inflammatory drugs (NSAIDs) or opioid analgesics. A non-disclosure agreement and a library of compounds soon followed. 

**Figure 2:**
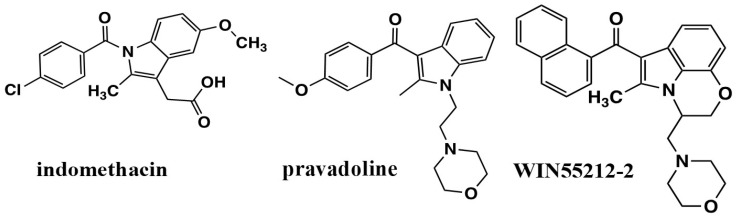
NSAID indomethacin, and aminoalkylindoles pravadoline and WIN55212-2.

Chemists from the Sterling Research Group were exploring non-steroidal anti-inflammatory analgesics, pravadoline, and its analogs, similar in structure to the well-recognized NSAID indomethacin (Figure 2). Pravadoline, comprised of an indole nucleus with an alkylamine substituent extending from the indole N, was a cyclooxygenase inhibitor like NSAIDS and blocked the formation of prostaglandins with a potency comparable to ibuprofen or naproxen, but less than indomethacin and more than acetaminophen [17]. Unlike these NSAIDs, pravadoline was an order of magnitude less potent in acute or chronic anti-inflammatory models and did not promote gastrointestinal ulcers in rodents [17]. Nevertheless, in a battery of seven antinociceptive tests in rodents, pravadoline exhibited potency that was comparable to aspirin and ibuprofen but less than indomethacin or naproxen. Pravadoline was less potent than morphine in these same antinociception tests; however, its effects could not be attributed to an opioid receptor because pravadoline’s response in the acetic acid-induced writhing test was not blocked by the opioid antagonist naloxone [17]. Other data not shown indicated that this response was also not due to serotonin receptors, α_1_- or α_2_-adrenergic receptors, or P_1_ or P_2_ purinergic receptors [17]. 

To address the mechanism of action, the Sterling Research Group found that pravadoline mimicked the opioid receptor-mediated relaxation of mouse vas deferens contractions, yet this response was not blocked by naloxone [17]. They chose three aminoalkylindole (AAI) analogs that were incapable of cyclooxygenase inhibition to test for their ability to inhibit guinea pig ilium and mouse or rat vas deferens contractions [18,19]. The analogs differed from pravadoline by being devoid of the (R)-α-methyl on the indole or having a naphthoyl group replace the aroyl [18]. The naphthoyl analogs were one to two orders of magnitude more potent than pravadoline at inhibition of electrically contracted vas deferens or guinea pig ileum, whereas prototypical NSAIDs had no effect [19]. These responses to pravadoline and its naphthoyl analog were not reversed by antagonists of mu, delta and kappa opioid, α1-adrenergic, P_1_-purinergic, or various serotonergic receptors [19]. Pravadoline and its naphthoyl analog failed to inhibit smooth muscle contractions in response to bradykinin or substance P, suggesting that the AAI effects were on presynaptic neurotransmiiter release. Interestingly, when various other neurotransmitter receptor agonists were tested, delta-opioid agonist peptide DADLE and the cannabinoid analgesic levonantradol were the most potent to inhibit vas deferens and guinea pig ileum contractions [19]. 

Additional AAI compounds were developed and evaluated using the mouse vas deferens and adenylyl cyclase assays. The naphthoyl AAI evoked inhibition of basal- and forskolin-stimulated adenylyl cyclase in rat brain cerebellar membranes in the presence of a cyclic nucleotide phosphodiesterase inhibitor [20]. For several analogs, the potency to inhibit adenylyl cyclase correlated with their potency to inhibit contractions in the mouse vas deferens [20]. This led to the discovery of a novel conformationally restrained enantiomeric pair in which a morpholinoethyl side chain was closed at position seven on the indole ring. This compound was given the code WIN55212-2 for the active (R) isomer and WIN55212-3 for the inactive (S) isomer (Figure 2) [20,21]. 

The development of AAI compounds also included an antagonist for the AAI agonists, WIN56098, which was created by the replacement of the C3-naphthoyl with a three-ringed anthracene. WIN56098 evoked competitive antagonism of the mouse vas deferens inhibition by pravadoline, the naphthoyl analog, and WIN55212-2, as well as inhibition of brain adenylyl cyclase by WIN55212-2 [20]. WIN56098 failed to compete in radioligand binding screens for α_1_-, α_2_-, β_1_-, β_2_-adrenergic, muscarinic and nicotinic cholinergic, H_1_ and H_2_ histamine, mu, delta and kappa opioid, 5-HT_1a–d_ and 5-HT_2_, NK-1 tachykinin, NMDA, phencyclidine, bombesin, and AngII receptors (Novascreen). Of a number of other neurotransmitter and neuromodulator agonists in the mouse vas deferens assay, the only non-AAI compounds that WIN56098 competitively antagonized were galanin, pargyline, Δ^9^-THC, and levonantradol [20]. WIN56098 has not achieved attention from the cannabinoid receptor research community, possibly because its log dose–response curve against WIN55212-2 exhibited a steeper slope than expected for a competitive antagonist [20] (A. Howlett, unpublished data), and it was not able to produce antagonism in vivo in rodent models of cannabinoid activity [22]. The Sterling Research Group also developed the antagonist 6-Br-pravadoline, which antagonized CB_1_-mediated inhibition of adenylyl cyclase at very low potency (>1 µM) (A. Howlett, unpublished data). 

Thus, armed with the knowledge that antinociceptive AAIs devoid of cyclooxygenase-inhibitory activity could produce in vitro responses resembling those of the cannabinoid agonists, it is not surprising that the Sterling Research Group would engage Dr. Howlett to screen a wide range of AAI compounds in her newly developed [^3^H]CP55940 radioligand binding assay for cannabinoid receptors. Dr. Howlett reported the final results to the Sterling Research Group in Spring 1990, providing evidence that AAI compounds displaced [^3^H]CP55940 from rat brain cannabinoid receptors over a wide range of IC_50_ values, with WIN55212-2 being the most potent and pravadoline being the least potent [23]. 

Simultaneously, the Sterling Research Group developed a radiolabeled [^3^H]WIN55212-2 for use in binding assays. They demonstrated that the potency of AAI compounds to compete for [^3^H]WIN55212-2 binding sites in rat cerebellar membranes correlated with inhibition of mouse vas deferens contractions [21,24]. Of the 60 neurotransmitter or neuromodulator agonists tested, none competed for [^3^H]WIN55212-2 binding except cannabinoid ligands [24]. The final evidence that the AAI analgesic compounds bind to brain cannabinoid receptors came from the development of an irreversibly binding isothiocyanato-desmethyl naphthalene AAI [25]. When this affinity ligand was used to pretreat rat brain membranes, its covalent binding depleted 90% of the [^3^H]CP55940 binding sites [25]. 

The greatest density of [^3^H]WIN55212-2 binding sites occurred in membranes prepared from the cerebellum, hippocampus, and striatum, with very little binding in the midbrain and spinal cord [24]. In studies of [^3^H]WIN55212-2 autoradiography in rat brain sections, the binding pattern was similar to that reported previously for [^3^H]CP55940 [26]. Studies in the mouse “tetrad” of cannabinoid-elicited behaviors (hypolocomotion, hypothermia, antinociception, catalepsy-like immobility) indicated that the naphthoyl AAI analogs that could inhibit the mouse vas deferens contractions were able to mimic Δ^9^-THC in vivo [22]. In addition, stereospecificity was demonstrated for the WIN55212 enantiomers in the “tetrad” behaviors. Functionally, drug discrimination studies indicated that rats trained to recognize Δ^9^-THC were able to identify the naphthoyl AAI analogs and the active enantiomer WIN55212-2 but not the inactive WIN55212-3 [22]. Important considerations in interpreting in vivo investigations include the pharmacokinetics and biotransformation of WIN55212-2. In a study published a decade later, Zhang and colleagues identified up to eight arene oxidative products following incubation with rat liver microsomes [27], which could have influenced biological activity. We can conclude that both common brain anatomic distribution patterns and behavioral similarities in rodent models demonstrate that the analgesic AAI compounds indeed bind to and stimulate the brain cannabinoid receptors. 

Sterling Winthrop, Inc. abandoned the AAI analgesic drug discovery project in June 1990 (personal communication S.J. Ward to A.C. Howlett). Some compounds were made available to researchers in collaborative projects, and Sterling Research Group scientists published their research findings to inform the biomedical research community of this novel class of AAI cannabimimetic compounds. Sterling Winthrop, Inc. formed a strategic alliance with the French pharmaceutical company Elf Sanofi in 1991, and the final acquisition of the Sterling Winthrop, Inc. prescription drug component by Elf Sanofi occurred in June 1994 [28].

## 2. Aminoalkylindoles and Cannabinoids: Structure—Activity Relationship Studies in Search of a Common Pharmacophore

Given the abilities of AAI ligands to displace [^3^H]CP55940 and cannabinoid ligands to displace [^3^H]WIN55212-2 in rat brain preparations, an obvious hypothesis to test was that the AAI ligands occupy the same binding pocket of the CB_1_ cannabinoid receptor, and further, that AAI ligands share a common pharmacophore with cannabinoid ligands. The common pharmacophore hypothesis was considered by a number of laboratories, each of which proposed models of homologous functionalities overlaying the structures of WIN55212-2 with a cannabinoid ligand. The Structure–Activity Relationship (SAR) studies of AAI compounds was evaluated to test these hypotheses and to establish principles for novel pharmacotherapeutic drug design. The most extensive series of compounds to assess AAI interaction with the CB_1_ receptor was developed by the Huffman laboratory. The synthesis and characterization of the Huffman series, as well as AAI compounds from other laboratories, have been comprehensively reviewed [29,30].

**Figure 3:**
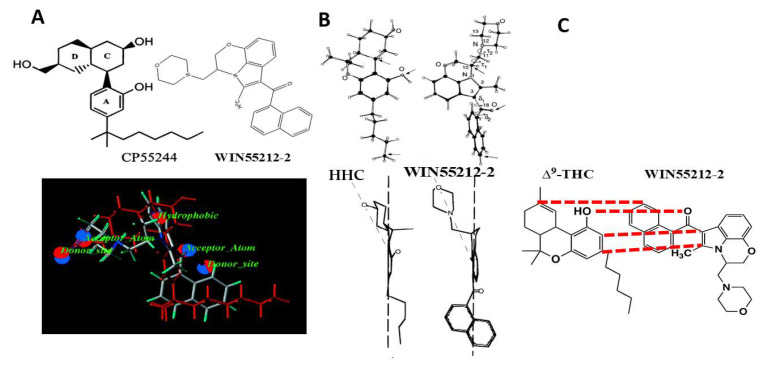
Alignments proposed for the common pharmacophore hypothesis for WIN55212-2 and cannabinoid agonists. (**A**) Alignment with CP55244, Shim and colleagues [31,35]. Reprinted with permission from Shim, J.Y. et al., J. Med. Chem. 45: 1447–1459, copyright 2002, American Chemical Society. (**B**) Alignment with (-)9β-OH-hexahydrocannabinol (HHC), Xie and colleagues [36,37]. Reprinted with permission from Xie, X.Q. et al., Life Sci. 56: 1963–1970, copyright 1995, Elsevier. (**C**) Alignment with Δ^9^-THC, Huffman and colleagues [29,38,39]. Redrawn using WIN55212-2, from Huffman Curr. Med. Chem. 1999 6: 705.

In St. Louis, computational chemists Welsh and Shim evaluated the Howlett data for the competition of AAI compounds with [^3^H]CP55940 binding in rat brain membranes [31,32]. These data became the training set for Comparative Molecular Field Analysis (CoMFA) to develop a 3D Quantitative SAR (QSAR) model based upon the steric and electrostatic fields surrounding the molecules in their protonated or non-protonated states. A parallel analysis was performed using K_i_ values from the Sterling Research Group, which reported competition of AAI compounds with [^3^H]WIN55212-2 binding in rat cerebellar membranes [33,34]. The resulting CoMFA models indicate that 80% of the variation in AAI ligand affinities for the CB_1_ receptor is based upon steric interactions. The potency of the AAI ligands to compete for the [^3^H]CP55940 binding site correlated well with their ability to act as agonists to inhibit hormone-stimulated adenylyl cyclase activity, with no evidence in the slope factors to suggest multiple receptors or cooperativity [32]. Based upon both the ligand-binding models and the requirements for agonist activity, it was proposed that the cannabinoid C3 side chain and the AAI C3 aroyl ring moiety both utilize hydrophobic interactions with residues within the CB_1_ receptor binding pocket. Further molecular modeling led to an alignment in which these moieties in CP55244 (the most potent and stereo-selective, A,C,D-tricyclic, non-classical cannabinoid of the Pfizer series) and WIN55212-2, respectively, were overlaid (Figure 3A) [31]. However, compelling data also indicated that AAI binding to the CB_1_ cannabinoid receptor might not result from the same chemical-binding interactions with receptor residues within a shared or overlapping binding pocket. This prediction was based upon evidence that the affinity of the AAI ligands for the [^3^H]CP55940 binding site was less than for the [^3^H]WIN55212-2 binding site (for six of the seven compounds assayed in both binding assays) [32]. Additional evidence was that the correlation was only “moderately strong” (r = 0.73) between the predicted K_i_ from the [^3^H]CP55940 binding model and the actual K_i_ from the [^3^H]WIN55212-2 binding experimental results [32], which is not supportive of identical ligand–receptor binding mechanisms within the shared binding pocket.

The Makriyannis and Xie laboratory, in collaboration with the Sterling Research Group chemist Eissenstat, used high-resolution 2D NMR with molecular modeling to develop a superimposition of the active enantiomeric structures of WIN55212-2 over the cannabinoid (-)9β-OH-hexahydrocannabinol (HHC) (Figure 3B) [36,37]. In this model, the AAI naphthoyl moiety overlaid the cannabinoid side chain, the WIN55212-2 fixed morpholino group overlaid the HHC cyclohexanol hydroxyl, and the AAI C3-carbonyl overlaid the cannabinoid phenolic hydroxyl [36]. They calculated the minimum energy conformations for the orientation of the naphthyl ring with respect to the carbonyl and of the morpholino group with respect to the C2-methyl in WIN55212-2 [37]. These studies determined a low-energy structure for WIN55212-2.

**Figure 4:**
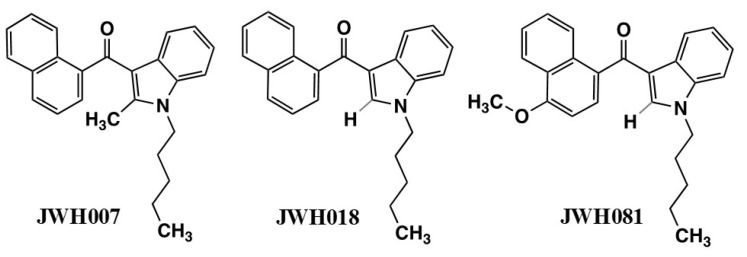
Alkyl indole compounds developed to test the Common Pharmacophore Hypothesis.

The Huffman laboratory proposed an alignment of WIN55212-2 with Δ^9^-THC by which the WIN55212-2 fixed morpholino moiety overlaid the cannabinoid C3-alkyl side chain; the AAI 3-carbonyl overlaid the cannabinoid phenolic hydroxyl, and the AAI naphthoyl group overlaid the Δ^9^-THC cyclohexene ring (Figure 3C) [29,38,39]. To test the role of the AAI N-ethylmorpholino of pravadoline and WIN55212-2, the Huffman group developed a series of indole and pyrrole analogs that were substituted at that position with alkyl chains of 1–7 carbon lengths, in the presence or absence of the C2-methyl (Figure 4) [38]. The most potent ligands to bind to the CB_1_ receptor [^3^H]CP55940 site also performed well in the behavioral “tetrad” tests and substituted for CP55940 in the drug discrimination trials. Properties of high potency ligands were: (1) N1-pentyl substituent; (2) no C2-methyl substituent; and (3) an indole rather than a pyrrole nucleus [38,39]. Potencies in all behavioral tests correlated well with the affinity for the displacement of [^3^H]CP55940 in rat brain membranes. Interestingly, the methyl, ethyl, and propyl pyrroles failed to bind to the cannabinoid receptor [^3^H]CP55940 site but exhibited behavioral responses of hypolocomotion, hypothermia, and antinociception, albeit with low potency or efficacy (see “What Additional Targets Exist for Aminoalkylindoles?”). Both Δ^9^-THC and WIN55212-2, were more potent at decreasing spontaneous activity than antinociceptive or hypothermic responses; however, the difference in potencies with WIN55212-2 was double that of Δ^9^-THC. 

In support of the Huffman model, the AAI aminoalkyl group could be replaced with alkyl substituents that resembled the cannabinoid C3 alkyl moiety. In order to assess whether the alignment was correct, the Huffman group synthesized a “hybrid” cannabinoid, JWH-161, in which the structure of Δ^9^-THC was fused to an indole nucleus having an N1-pentyl substituent [40]. JWH-161 exhibited potencies for [^3^H]CP55940 binding to the CB_1_ receptor and cannabimimetic “tetrad” tests that were comparable to those of Δ^9^-THC. Although this result is consistent with the region of the cannabinoid C3 alkyl side chain interacting with the receptor via hydrophobic interactions, it does not necessarily invoke the necessity of an indole nucleus in this binding domain. The Huffman model aligns the AAI indole carbonyl moiety with the cannabinoid phenolic hydroxyl, which is required for cannabinoid agonist activity at the CB_1_ receptor. Removal of the AAI indole carbonyl in naphthylidene indene conformers (E active versus Z inactive) reduced affinity for the [^3^H]CP55940 binding site [41]. The reduced affinity was calculated to be due to the modification of the linkage angles and orientation of the aryl ring structure [42], which overshadowed the assessment of a potential role for oxygen in hydrogen-bonding interactions. 

In a series of N1-ethylmorpholino, 3-naphthyl indoles devoid of the carbonyl oxygen, K_i_ values for [^3^H]CP55940 binding displacement were in the 40–42 nM range [42]. For their N1-pentyl analogs, also devoid of carbonyl substituents, K_i_ values were in the 17–23 nM range [42]. JWH-176, an indene molecule devoid of oxygen or nitrogen atoms, exhibited a K_i_ = 26 nM. These affinities compare favorably with the K_i_ = 10 nM reported for WIN55212-2 in the same data set. These data favor the dominance of aromatic stacking interactions with very little influence of hydrogen bonding for AAI interactions with the CB_1_ cannabinoid receptor. 

To assess the CB_1_ cannabinoid receptor agonist binding requirements, it was known that mutation of a transmembrane helix-3 lysine to alanine in the hCB_1_ receptor expressed in HEK293 cells conflicted with competition for [^3^H]WIN55212-2 by cannabinoid ligands but not by WIN55212-2 [43]. The potency of cannabinoid agonists to inhibit cAMP production was reduced in cells expressing the mutant receptors, but the response to WIN55212-2 was unaffected. These findings suggest that the required phenolic hydroxyl on cannabinoid structures was hydrogen bonding with this lysine but that this hydrogen-bonding interaction was not a factor in the AAI interactions. In contrast, when CB_1_ receptor mutants of a highly conserved helix-2 aspartate were expressed in HEK293 cells, cannabinoid agonist displacement of [^3^H]CP55940 was not affected, but WIN55212-2 binding suffered a 45-fold reduction in affinity when the aspartate was mutated to asparagine, and an 8.5-fold reduction in affinity when mutated to glutamate [44]. These findings suggest that this helix-2 aspartate must be involved in WIN55212-2 but not cannabinoid agonist interactions. 

To identify the CB_1_ cannabinoid receptor mechanism for AAI ligand binding, the Reggio group developed a homology model based upon the structure of activated rhodopsin [41,42]. The conformation of WIN55212-2 and pravadoline as S-trans (versus inactive S-cis) within the activated cannabinoid receptor binding pocket was predicted by pharmacological results demonstrating the preferred conformation of rigid naphthylidene indene analogs of AAIs to exist as the active “E” (comparable to S-trans) as opposed to the “Z” (comparable to S-cis) conformation [41].

The Reggio group reported that an aromatic cluster of residues in transmembrane helices 3, 4, and 5 are a likely binding pocket to accommodate hydrophobic ligand interactions [45,46]. Using the rhodopsin homology model in the “active state”, residues that include helix-3 phenylalanines and helix-4 and helix-5 tryptophans could form an aromatic stack that is energetically favored [46]. A hydrophobic binding pocket of helix-3 valine, isoleucine, and phenylalanine, and helix-6 leucine and isoleucine could accommodate an alkyl chain between three and six carbons in length, and helix-5 and helix-6 tryptophans could allow aromatic stacking interactions with the indole and naphthyl moieties [42]. With this configuration, the binding energy would be due to hydrophobic interactions, although as a minor contribution, a hydrogen bond could exist between N–H of the helix-5 tryptophan and the carbonyl oxygen. This hydrogen bond would not be possible for the indene analogs lacking oxygen and was suggested to be responsible for their reduced potency [42].

Shim and Howlett addressed the mechanism by which WIN55212-2 could trigger a response to activate the CB_1_ receptor [47]. Using a homology model based on rhodopsin in the inactive “ground” state, Shim performed Monte Carlo and molecular dynamics simulations to identify the docking conformations exhibiting the lowest ΔE_bind_ values for WIN55212-2 within the CB_1_ receptor binding pocket [47]. They correlated the calculated docking ligand–receptor interaction energy with experimental binding affinity data for 37 AAI compounds to compete for [^3^H]WIN55212-2 binding sites in rat brain membranes under basal conditions (the absence of Na^+^ or GTP analogs) [33]. Two conformations having the greatest correlation were identified as having the aroyl groups oriented “up” closest to the extracellular surface of the receptor in the hydrophobic binding space. The interaction energies with amino acids within 3 Å were identified as predominantly van der Waals (steric), with minor contributions of electrostatic (i.e., ionic or hydrogen-bonding) forces, in agreement with previous studies (discussed above). It was hypothesized that the WIN55212-2 structure docked in the ground state would be able to exert a “trigger” to induce one or more micro-conformational changes essential for the process of CB_1_ receptor activation. Strain energy is released as the agonist bound to the receptor relaxes to achieve its lowest energy conformation. The energy released from the conformational change in the agonist ligand is the driving force for inducing conformational changes in the receptor that is necessary for transferring the signal to G-proteins. To determine how this might occur, Shim determined the “flexibility” of four torsion angles of the WIN55212-2 molecule to identify intrinsic changes in the agonist’s conformations after being bound to the ground state of the CB_1_ receptor. In molecular dynamics simulations in the absence of the receptor, a conversion from S-*trans* to S-*cis* could occur as the torsion angle between the carbonyl oxygen and the naphthoyl ring adjusts to reduce the steric repulsion to the indole ring. This allows WIN55212-2 to traverse the lowest possible rotational energy barrier within the allowed conformational space. As the ligand conformation “switches” to release strain energy and attain the lowest possible energy conformation, this “switch” becomes the “steric trigger” to allow WIN55212-2 to force a change in the receptor conformation. If the lowest energy conformation of the agonist creates an unfavorable steric clash with amino acids within the receptor hydrophobic pocket, then the receptor adjusts its conformation. This may occur as series of micro-conformational changes to ultimately achieve the activated state. Conceivably, different ligand-binding conformations for the same binding pocket may initiate diverse types of receptor motions for ligand-specific conformational changes within the receptor. Thus, it is not likely that the AAI [47] and cannabinoid [48,49] agonists utilize the same “mechanism” to trigger micro-conformational changes to activate the CB_1_ receptor. 

In total, these studies have identified a pharmacophore for AAI ligands to bind within a hydrophobic pocket of the CB_1_ receptor. AAI binding overlaps within the binding pocket for cannabinoid ligands. However, the interactions with amino acids and the mechanism for activation of the receptor differ, resulting in subtle conformational differences that could result in selective interactions with their transducers (G proteins, β-arrestins, other associated proteins).

## 3. The Quest for Selective CB_2_ Cannabinoid Receptor Ligands

One of the challenges to cannabinoid pharmacology has been the separation of agonist activities for the CB_2_ versus the CB_1_ cannabinoid receptors. A highly selective CB_2_ agonist would be useful as an anti-hyperalgesic and anti-allodynic agent in neuropathic as well as anti-inflammatory pain [50,51,52]. The requirements for an ideal CB_2_ pharmacotherapeutic agent are (1) to function with high potency and efficacy at the CB_2_ receptors, but also (2) to have low affinity for the CB_1_ receptors that stimulate untoward central nervous system effects such as sedation and cognitive and memory dysfunction. Evidence based upon the preclinical studies of Huffman and multiple pharmaceutical researchers suggests that the challenge might be met with AAI compounds (reviewed in [53,54,55]).

### 3.1. CB_2_-Selective Indole Agonists

#### 3.1.1. JWH-015 and Analogs (1-Propyl-2-methyl-3-(1-naphthoyl) indole)

The first observation of cannabimimetic indoles showing CB_2_ receptor selectivity was that WIN55212-2 exhibited greater affinity in [^3^H]CP55940 binding in stably expressing hCB_2_-Chinese Hamster Ovary (CHO) fibroblastic cells compared with hCB_1_-CHO cells [56]. In an effort to identify additional CB_2_-selective ligands, the Abood laboratory examined [^3^H]CP55940 binding in hCB_2_-CHO or hCB_1_-CHO cells [57]. WIN55212-2 exhibited a 7-fold selectivity for the CB_2_ receptors, but because WIN55212-2 was quite potent at binding to both receptor types, it exhibited potent CB_1_-mediated effects in the behavioral “tetrad” tests, which would make it unlikely to serve as a selective CB_2_ receptor agonist. The other AAI that showed CB_2_-selectivity was JWH-015, which exhibited greater than 25-fold selectivity for binding to the CB_2_ receptor [57]. JWH-015, a propyl analog of pravadoline, exhibited very low affinity at the CB_1_ receptor and relatively low potency in the behavioral “tetrad” behaviors [57]. It is interesting to note that the slope of the log dose–response [^3^H]CP55940 binding curve for JWH-015 was shallower than expected for a single binding site, which could indicate either binding to two different receptors, binding to two different affinity states of the CB_2_ receptor, or negative allosteric regulation of the CB_2_ receptor. This interesting phenomenon has yet to be explained in the research literature.

**Figure 5:**
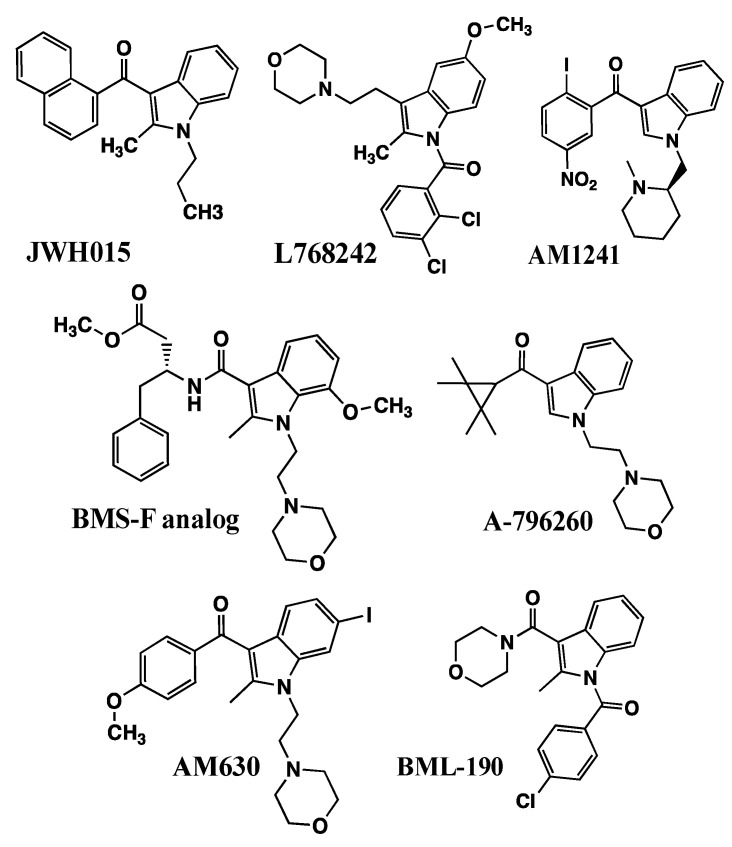
CB_2_-selective indole ligands.

JWH-015 is a member of a series of C3-naphthoyl indoles in which a propyl substituent was appended to indole N1 (Table 1 and Figure 5). Although the propyl analog reduced the ability to bind to the CB_1_ receptor compared with the pentyl analog, it nevertheless retained behavioral “tetrad” activities [38]. Among C3-naphthyl indole analogs lacking the C2 methyl, the N1 alkyl chain length correlated with [^3^H]CP55940 binding affinity in hCB_2_-CHO membranes, increasing nearly 20-fold in going from ethyl to a propyl, whereas the CB_1_ receptor binding in rat brain membranes remained at nearly the same poor affinity [58]. The propyl analog, JWH-072, yielded a CB_1_/CB_2_ selectivity ratio = 6. Both CB_1_ and CB_2_ receptor binding reached maximal potencies at butyl, pentyl, and hexyl, at which the CB_2_/CB_1_ selectivity ratio was reduced to ≤3. 

Because the addition of a C2-methyl reduced affinity for CB_1_ receptors [38], it was observed that a methyl modification in the propyl analog JWH-015 improved CB_2_/CB_1_ selectivity ratio = 24. Selectivity was not improved by adding a C7′-methyl substituent onto the naphthoyl ring system in JWH-046 (CB_2_/CB_1_ selectivity ratio = 21) [58], but it was encouraging that for JWH-046, maximal activities in the cannabimimetic “tetrad” tests could not be attained [38]. These compounds (Table 1) were agonists in CHO-CB_2_ membranes in the [^35^S]GTPγS binding assay of G protein activation, with JWH-151 showing full efficacy compared with CP55940, and the others having partial agonist activity ranging from 65% to 80% compared with CP55940 [59]. An additional series of halogenated naphthoyl indoles was developed, three of which exhibited optimal high affinity for the CB_2_ receptor and a good CB_2_/CB_1_ selectivity ratio: JWH-423 (1-propyl-3-(4-iodo-1-naphthoyl)indole), JWH-422 (the 2-methyl analog of JWH-423), and JWH-417 (1-pentyl-3-(8-iodo-1-naphthoyl)indole) [60]. 

Development of the Huffman compounds promoted the recognition by leading drug companies that CB_2_ receptor selectivity could be achieved. It seemed that nearly half of the participants in the 2005 International Cannabinoid Research Society meeting were pharmaceutical industry scientists. The potential that CB_2_-selective indole cannabimimetics could be developed as anti-hyperalgesic and anti-allodynic medicines inspired tremendous interest in pharmaceutical companies to engage in preclinical studies (examples follow). 

#### 3.1.2. L768242/GW405833 (1-(2,3-Dichlorobenzoyl)-2-methyl-3-(2-[1-morpholine] ethyl)-5-methoxyindole)

The Merck Frosst Centre for Therapeutic Research reported that L768242, also known as GW405833 (Figure 5), exhibited a high affinity for the CB_2_ receptor (K_i_ = 14 nM) and a high CB_2_/CB_1_ selectivity ratio = 146 [61]. Valenzano and the Purdue Pharma Discovery Research group determined that L768242/GW405833 interacts with human CB_2_ receptor [^3^H]CP55940 sites in hCB_2_-CHO cells with high affinity (K_i_ = 3.9 nM) and a hCB_2_/hCB_1_ selectivity ratio = 1217 [62]. The affinity was the same for CB_2_ binding in rat spleen membranes (K_i_ = 3.6 nM), and comparison with rat brain membranes yielded a CB_2_/CB_1_ affinity ratio = 76. In the CB_2_-CHO cells, L768242/GW405833 was a partial agonist, exhibiting 50% efficacy compared with CP55940 to inhibit forskolin-stimulated cAMP accumulation [62]. 

Clayton and colleagues at Glaxo Wellcome Research and Development noted that L768242/GW405833 inhibited carrageenan-induced paw inflammation and hypersensitivity, and these effects were blocked by the CB_2_ antagonist SR144528 [63]. Valenzano and colleagues determined that L768242/GW405833 attenuated mechanical hyperalgesia in rat spinal nerve ligation or the rat paw incision tests but had no effect on thermal antinociception (tail-flick or hotplate tests) [62]. In the mouse paw chronic inflammation (Freund’s complete adjuvant) model, tactile allodynia was partially reversed, comparable in efficacy to indomethacin. The L768242/GW405833 response was not observed in CB_2_^−/−^ mice, but the indomethacin response was not tested (or reported) in the CB_2_^−/−^ mice. Beltramo and colleagues at Schering-Plough Research Institute reported that L768242/GW405833 was effective in neuropathic pain tests in rodents in which it attenuated hyperalgesia in the mouse intraplantar formalin model and allodynia in the rat spinal nerve ligation model [64]. Both responses were precluded by pretreatment with the CB_2_ antagonist SR144528.

#### 3.1.3. AM1241 ((R-) or (S-) 3-(2-Iodo-5-nitrobenzoyl)-1-(1-methyl-2-piperidinylmethyl)-1H-indole)

AM1241 (Figure 5) displaced [^3^H]CP55940 with two orders of magnitude greater potency in mouse spleen homogenates (abundant in CB_2_ receptors) compared with rat brain synaptosomal membranes (abundant in CB_1_ receptors) [65]. Bingham and colleagues at Wyeth Research identified two isomers: R (+) was two orders of magnitude more potent than S (−) to compete for [^3^H]CP55940 binding to human, rat, and mouse CB_2_ compared with CB_1_ receptors expressed in CHO cells [66]. Their investigation of forskolin-stimulated rCB_2_-CHO cells showed that S-AM1241 inhibited cAMP production, resembling WIN55212-2. In contrast, R-AM1241 augmented forskolin-stimulated rCB_2_-CHO cAMP production, resembling SR144528. Enantiomeric response differences between rodent and human CB_2_ receptors were complex [66] but might be influenced by the degree of “constitutive” activity in these exogenously expressed systems [67], the serum levels or cellular production of endogenous endocannabinoids, or differential sensitivity to endocannabinoids. 

In in vivo models of spinal nerve ligation in rats or mice, AM1241 (ip) dose-dependently attenuated both tactile and thermal hyperalgesia, both of which were antagonized by CB_2_-selective AM630 but not by CB_1_-selective AM251 [65]. Additional evidence against a CB_1_ involvement in the anti-hyperalgesic responses was that AM1241 effects were also observed in CB_1_^−/−^ mice. AM1241 attenuated carrageenan-induced inflammatory thermal hyperalgesia when injected directly into the inflamed paw but failed to evoke antinociception in the contralateral control paw [68]. In that model, AM1241 also reversed the local edema, and both edema and hyperalgesia responses to AM1241 were antagonized by AM630 but not AM251. 

Beltramo and colleagues showed that AM1241 could attenuate both hyperalgesia in mouse intraplantar formalin and allodynia in the rat spinal nerve ligation tests, and that both responses were inhibited by CB_2_-selective SR144528 [64]. S-AM1241 (but not R-AM1241) was as efficacious as indomethacin at prolonging the latency to remove a carrageenin-inflamed paw from a thermal stimulus [66]. The response to S-AM1241was reversed by CB_2_ antagonist AM630, but it was not determined if the response to indomethacin could also be reversed by AM630 or SR144528 [66]. 

#### 3.1.4. BMS Series and A796260 from 1-Alkyl-3-keto Indole Series

Bristol-Myers Squibb researchers developed a series of compounds based on a substituted indole 3-carboxylic acid nucleus (Figure 5) [69]. Their most promising compound was a phenylalanine-derived amide that exhibited high CB_2_ receptor affinity (K_i_ = 8 nM) and a very high CB_2_/CB_1_ affinity ratio = 500. 

Abbott researchers developed a series of 1-alkyl-3-keto indoles having variations in nitrogen side chains, with saturated cyclic ketones as the C3-aryl substituent. They identified A796260 (Figure 5) having a C3-tetramethylcyclopropyl substituent, as exhibiting extremely high affinity for the CB_2_ receptor expressed in CHO cells (K_i_ = 0.77 nM), an extremely high CB_2_/CB_1_ selectivity ratio = 2700, and full agonist efficacy in cellular functional assays [70]. A796260 was efficacious in in vivo models of chronic inflammatory pain and chronic neuropathic pain, and its responses were selectively blocked by CB_2_ antagonist, but not by CB_1_ or μ-opioid antagonists.

In aggregate, these studies identify local, CB_2_-dependent, anti-hyperalgesic and anti-allodynic responses in chronic inflammatory and neuropathic pain models that do not require a CB_1_ receptor involvement. These promising preclinical experimental results warrant further development in clinical settings. Even so, the cellular and biochemical mechanism of action of these compounds may not be entirely attributable to their actions at the CB_2_ receptor. For example, these compounds are analogs of pravadoline, an NSAID exhibiting antinociceptive actions that could be attributed to inhibition of prostaglandin synthesis. Complete understanding of the mechanism of action and potential for untoward side effects will require a more comprehensive investigation of the synthesis of anandamide in the pain process alleviated by these compounds, the contribution of anandamide to the “constitutive” activity of the CB_2_ receptor, and the contribution of these CB_2_-selective cannabimimetic indoles to the inhibition of COX2 in the inflamed tissue. 

### 3.2. CB_2_-Selective Indole Antagonists

#### 3.2.1. AM630 6-Iodo-Pravadoline

AM630 (6-iodo-pravadoline) (Figure 5) appears to respond either as an agonist or as a competitive antagonist and inverse agonist in different types of cell signaling determinations. AM630 was first identified to be a competitive antagonist in the cannabinoid inhibition of mouse vas deferens twitch response, right-shifting the log dose–response curves to Δ^9^-THC, CP55940, and WIN55212-2 (K_inh_ values were calculated to be in the 14 nM–36.5 nM range), but not to morphine or clonidine [71]. This report was followed by the determination that AM630 behaved as a low-potency agonist (IC_50_ = 1.9 µM) compared with WIN55212 (IC_50_ = 5.5 nM) to inhibit contractions of the guinea pig ileum [72]. These AM630 log dose–response curves were right-shifted by the CB_1_ antagonist SR141716, demonstrating AM630 to be a CB_1_ receptor agonist [72]. At high concentrations (100 µM), AM630 behaved as a competitive antagonist to right-shift the WIN55212-2-stimulated [^35^S]GTPγS binding curves in mouse [73] or guinea pig [74] brain homogenates (assumed to be abundant in CB_1_ receptors). In a CB_1_-CHO cell [^35^S]GTPγS binding determination, AM630 behaved as an inverse agonist to inhibit basal by 20% (EC_50_ = 900 nM), under the same conditions that WIN55212-2 behaved as an agonist to stimulate basal activity (EC_50_ = 360 nM) [75].

To clarify the activity of AM630 at the molecular level, Ross, Pertwee, and colleagues used CB_1_-CHO and CB_2_-CHO cell comparisons to determine affinity to displace [^3^H]CP55940 and activity for the cannabinoid receptors [76]. As the Pertwee lab had suspected from the studies in tissue preparations, AM630 interacted potently with the CB_2_ receptor (K_i_ = 31 nM) and exhibited a CB_2_/CB_1_ selectivity ratio = 165. AM630 behaved as a potent (EC_50_ = 76.6 nM) inverse agonist to inhibit basal [^35^S]GTPγS binding in CB_2_-CHO membranes; using the Landsman data in CB_1_-CHO cells, this yields a CB_2_/CB_1_ potency ratio approaching 12. Consistent with these data on G protein activation, AM630 at high concentrations (1 µM) behaved as an inverse agonist in CB_2_-CHO cells by augmenting forskolin-stimulated cAMP accumulation. In CH_2_-CHO cells, AM630 also behaved as a competitive antagonist for CP55940-Gi-mediated inhibition of forskolin-stimulated cAMP accumulation [76]. In contrast, in CB_1_-CHO cells, AM630 at high concentrations (1–10 µM) behaved as an agonist in Gi-mediated inhibition of forskolin-stimulated cAMP accumulation but exerted a tendency to attenuate the Gi-mediated agonist response to CP55940, making AM630 a partial agonist [76]. The Mackie laboratory found that in mCB_2_-HEK293 cells, AM630 behaved as an inverse agonist in cAMP production assays but behaved as a low-efficacy agonist in β-arrestin recruitment assays [77].

#### 3.2.2. BML190

BML-190 (Figure 5) has a low affinity for CB_2_ receptors exogenously expressed in CHO cells. BML190 appears to be an inverse agonist for the CB_2_ receptor, as it augmented forskolin-stimulated cAMP production [61].

### 3.3. CB_2_-Selective WIN55212-2 and AAI Ligand Interactions with the CB_2_ Receptor

As described for the CB_1_ receptor, the CB_2_ receptor engages cannabimimetic indoles via aromatic stacking mechanisms. However, the specific molecular interactions of CB_2_-selective AAI ligands with the CB_2_ receptor appear to differ from CB_1_-selective AAI interactions with the CB_1_ receptor.

The importance of amino acids in the CB_2_ helix-3 for AAI interactions was reported by Chin and Kendall, who created a chimeric CB_1_ receptor possessing the CB_2_ helix-3 and expressed the receptors in CHO cells [78]. The affinities for WIN55212-2 (K_d_ = 4.8 nM) and JWH-018 (K_d_ = 1.4 nM) were greater for the CB_2_-helix 3 chimera than for the CB_1_ receptor; however, JWH-015 (K_d_ = 1 µM) exhibited low affinity but still greater than for the CB_1_ receptor [78]. The average CB_2_-helix 3 chimera/CB_1_ selectivity ratio was 5.6. These affinities paralleled the potencies to inhibit cAMP accumulation in CHO cells expressing these receptors [78]. When individual amino acid differences were investigated by site-directed mutagenesis and expression in CHO cells, it appeared that the serine unique to the CB_2_ helix-3 was important for the WIN55212-2 interaction with cannabinoid receptors [78].

The Abood laboratory compared responses of CB_2_ to CB_1_ receptors expressed in HEK293 cells [79]. For the CB_1_ receptor, the helix-3 lysine192 was required for cannabinoid ligand binding but not WIN55212-2 binding. In contrast, when the comparable CB_2_ lysine109 was mutated to alanine, there were no differences from wild-type CB_2_ in cannabinoid or WIN55212-2 binding or agonist responses to inhibit cAMP accumulation [79]. However, the CB_2_ helix-3 serine112 mutation to glycine double mutant with the lysine109 mutation to alanine compromised the cannabinoid agonist but not WIN55212-2 binding [79]. 

Interestingly, there are two reports of loss of cannabinoid ([^3^H]HU243 and [^3^H]CP55940) as well as [^3^H]WIN55212-2 binding resulting from mutation of the CB_2_ receptor helix-3 aspartate that is part of the “DRY” sequence and a coordinating helix-6 alanine [80,81]. Because both amino acids affecting CB_2_ receptor binding are located at the intracellular juxtamembrane surface, it is likely that their influence is on rigid helical movement or conformational modifications transmitted along the helices that would affect interactions with the ligands occurring near the extracellular membrane surface. 

Several investigations were reported to test the hypothesis that aromatic stacking is important for WIN55212-2 interaction with the CB_2_ receptor. Interaction of WIN55212-2 with a phenylalanine in helix-5 unique to the CB_2_ receptor was predicted by the Reggio laboratory using in silico docking models [46]. When tested with site-directed mutagenesis and expression in HEK293 cells, the CB_2_ receptor mutation of phenylalanine to valine compromised the affinity for WIN55212-2 but did not affect the affinity for cannabinoid ligands HU210 or CP55940 [46]. Parallel changes in the ability to inhibit cAMP accumulation were observed in these cells. A conserved helix-5 tyrosine, important for aromatic stacking in both CB_1_ and CB_2_ receptors, was necessary for stimulation of signaling by both WIN55212-2 and cannabinoid agonists [45]. Two CB_2_ helix-4 tryptophans (or their conservative mutation to phenylalanine) were essential for [^3^H]HU243 binding and for HU210- or WIN55212-2-mediated inhibition of cAMP production in hCB_2_-COS7 cells [82]. 

Structural interactions between CB_2_ receptors and the AAI ligands compared with cannabinoid ligands can lead to functional differences (biased agonism) as demonstrated by the Mackie laboratory for rodent CB receptors expressed in HEK293 cells [77,83]. For example, CP55940 was a full agonist in CB_2_-Gi-mediated inhibition of cAMP production, whereas WIN55212-2 had lower efficacy [77]. Both WIN55212-2 and CP55940 recruited β-arrestins to the plasma membrane, whereas classical cannabinoid and most AAI ligands failed [77,83]. CP55940 and cannabinoid ligands promoted the internalization of CB_2_ receptors, whereas WIN55212-2 and other AAI ligands did not [83]. The functional selectivity, very likely based upon conformational differences in the structural mechanisms of activation of the receptors by the ligands, can initiate cellular signaling pathways that are uniquely different in target cells. Thus, conflating the cellular responses to cannabimimetic indoles with responses to classical cannabinoids such as Δ^9^-THC can lead to misrepresentation of physiological and pharmacological endpoints. 

## 4. What Additional Targets Exist for Aminoalkylindoles?

### 4.1. Non-CB_1_, Non-CB_2_ Targets for WIN55212-2

Early in the investigation of WIN55212-2’s binding and cellular-signaling properties, Childer’s laboratory recognized that displacement of [^3^H]WIN55212-2 binding by cannabinoid ligand CP55940 differed between rat brain cerebellar membranes (IC_50_ = 1.2 nM) and cultured mouse neuroblastoma–rat glioma hybrid cell NG108-15 membranes (IC_50_ > 5000 nM) [84]. The properties of the binding site in cerebellar membranes were typical of a GPCR in that binding affinity for the agonist [^3^H]WIN55212-2 was reduced by GTPγS or by Na^+^, whereas those binding sites in the hybrid cell were resistant to these regulators. These data suggest that the binding sites were not the same and that only those binding sites in the cerebellar membranes were GPCRs. With the advent of modern molecular biology techniques, the neuroblastoma–glioma hybrid cell line lost its popularity due to its polyploidy, which in fact allows the NG108-15 hybrid cells to express both rat and mouse mRNAs for the CB_1_ receptor [85]. The Howlett laboratory determined that the NG108-15 cell line was capable of stimulating a functional inhibition of adenylyl cyclase in membrane preparations, albeit with less response than in membranes from the N18TG2 neuroblastoma parent, and that membranes from the rat C6-glioma parent fail to respond to cannabinoid ligands [13,86]. Thus, although [^3^H]WIN55212-2 fails to recognize these low-abundance functional CB_1_ receptors in the NG108-16 cells, this ligand recognizes an alternative protein target that binds extremely poorly to CP55940 [84] (and perhaps other cannabinoid ligands as well).

If the only target in the brain for WIN55212-2 were the CB_1_ receptor, then that target should not be present in the CB_1_^−/−^ mouse brain. Breivogel and colleagues performed this test in a study of [^35^S]GTPγS binding to activated G proteins in brain membranes from the C57Bl/6 CB_1_^−/−^ mouse as ablated by Zimmer and colleagues [87]. They demonstrated that the knock-out of CB_1_ receptors resulted in a loss of the response to high-efficacy cannabinoid agonists CP55940 and HU210 as well as partial agonist Δ^9^-THC [87]. However, anandamide and WIN55212-2 both evoked a response in CB_1_^−/−^ mouse brain membranes. Estimates of SR141716-resistant stimulation in wild-type mouse brain membranes suggested that 16% of the anandamide- and 33% of the WIN55212-2-stimulated response might be due to non-CB_1_ target(s) [87]. The WIN55212-2-stimulated response in the CB_1_^−/−^ mouse brain was localized to regions that in wildtype mice do not express an abundance of CB_1_ receptors (brainstem, diencephalon, midbrain, and spinal cord), whereas the WIN55212-2 response was not significantly stimulated in regions expected to express high densities of CB_1_ receptors (basal ganglia, cerebellum) [87]. These same findings were reported for the CD1 CB_1_^−/−^ mouse ablated by Ledent and colleagues, with some discrepancies in brain regions expressing the response [88]. In their investigation, anandamide and WIN55212-2 were not able to inhibit adenylyl cyclase, suggesting that the novel WIN55212-2-stimulated target does not couple to Gi proteins [88]. 

Neurophysiological investigations provided additional evidence for a non-CB_1_ WIN55212-2 target in the brain. In the mouse hippocampus, which exhibits a well-characterized, CB_1_-mediated suppression of neurotransmission at GABAergic presynaptic terminals, Hájos, Ledent and colleagues found that WIN55212-2 compromised neurotransmission at glutamatergic synapses in both wild-type and Ledent CD1 CB_1_^−/−^ mice [89,90]. They recognized a high-affinity (nM range), CB_1_-mediated reduction in Schaffer collateral-evoked CA1 pyramidal cell excitatory post-synaptic potentials in rat brain slices. However, they also identified a low-affinity suppression of neurotransmission response to WIN55212-2 (µM range) in brain slices pretreated with CB_1_ antagonist AM251 [90]. This non-CB_1_ response was blocked by pretreatment with Ω-conotoxin GVIA, suggesting that WIN55212-2 might directly target N-type, voltage-gated Ca^2+^ channels or work via a GPCR that targets the N-type channels [90].

WIN55212-2 (µM range) inhibited the frequency of rat nucleus tractus solitarius glutamatergic and GABAergic stimulated postsynaptic currents [91]. This response was not observed with cannabinoid agonist HU210 or CB_1_-selective agonist arachidonyl cyclopropylamide. The WIN55212-2 response could not be blocked by CB_1_ antagonist AM251, CB_2_ antagonist AM630, or TRPV1 blocker AMG9810, suggesting that an alternative target is responsible [91]. Because the nucleus tractus solitarius receives direct inputs from cardiovascular reflex detectors, this novel WIN55212-2 target might disrupt autonomic baroreflex regulation of blood pressure. 

### 4.2. Putative Alkyl Indole Receptors

**Figure 6:**
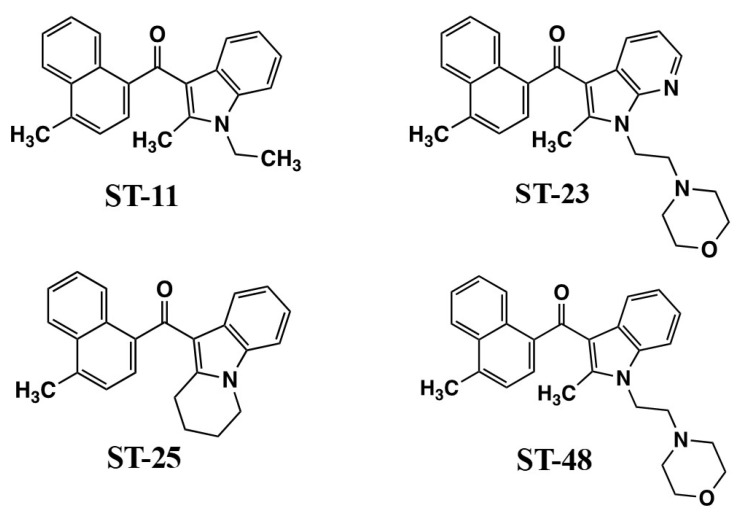
The Stella naphthoyl indoles and analogs.

The Stella laboratory discovered that WIN55212-2 might be acting at brain microglia cell targets via a non-CB_1_, non-CB_2_ mechanism [92]. In order to characterize the responsible receptor, which they termed the Alkyl Indole (AI) receptor, they developed analogs that could distinguish the novel AI functions [93,94]. ST-11 and ST-48 (Figure 6) are naphthoyl indoles that exhibit high affinity for [^3^H]WIN55212-2 binding sites (32.6 nM, 23.7 nM, respectively) in membranes from primary cultures of mouse microglia [93,94]. AI receptor stimulation by ST-11 promoted cAMP accumulation and inhibited both basal migration as well as ATP-driven chemokinesis in a Boyden chamber test [93]. ST-11 also inhibited macrophage-colony-stimulating-factor-induced proliferation but did not alter responses to cytokines that direct the determination of microglia to develop M1 (pro-inflammatory) or M2 (anti-inflammatory) phenotypes [93]. However, differentiation to an M2 phenotype was sufficient to attenuate the responses to ST-11, demonstrating that signaling by the AI receptors is subject to modulation by other ongoing cellular signal transduction pathways.

Previous studies indicated that certain non-CB_1_ effects of WIN55212-2 did not appear to involve GPCRs. In the course of investigating ST-11 and its analogs, the Stella laboratory discovered the ability of ST-11 to reversibly interact with the colchicine-binding site of microtubules and attenuate their assembly [95]. In fast-growing glioblastoma tumor cells, this led to disruption of spindle formation, cell cycle arrest in pro-metaphase, and subsequent apoptosis [95]. This response makes ST-11 of great clinical significance as a potential cancer chemotherapeutic agent for glioblastoma. Unlike many mitosis-disrupting cancer drugs, ST-11 avoids multi-drug resistance pumps, and gains access to the brain through the blood–brain barrier when formulated in lipid nanodiscs for efficient delivery [95]. 

Further drug development to identify the cellular role of AI receptors required a separation of AI activation from microtubule-binding properties in addition to CB_1_ and CB_2_ cannabinoid receptors. ST-11 fails to bind to CB_1_ and CB_2_ receptors and exhibits an AI/colchicine binding selectivity ratio = 61.5, which makes it possible to access the brain at concentrations that favor AI receptor-mediated responses [94]. Using a model of DBT cells, which do not express CB_1_ or CB_2_ mRNA or [^3^H]CP55940-binding sites, the Stella team demonstrated that the [^3^H]WIN55212-2 binding site recognized WIN55212-2 (K_i_ = 6.2 nM) and ST-11, ST-23, ST-25, and ST-48 (K_i_’s in the 21 nM–52 nM range) (Figure 6), but not CB_1_ antagonist SR141716, CB_2_ antagonist SR144528, or an inactive indole ST-47 [94]. ST-11, ST-25, and ST-48 were agonists to inhibit basal- and lysophosphatidic acid-mediated chemokinesis, with ST-48 having the greatest potency (EC_50_ = 5 nM). ST-23, ST-25, and ST-48 at high concentrations (3 µM) promoted internalization of HA-mCB_1_ (but not HA-mCB_2_) receptors expressed in HEK293 cells. ST-11 and ST-29 at high concentrations (3 µM) competed for [^3^H]colchicine binding to tubulin preparations [94]. Thus, there is evidence for functional selectivity within this series of AI ligands, with AI receptors regulating cellular signaling at nM concentrations while avoiding off-target properties such as CB_1_-binding and tubulin disruption that occur at high concentrations that might not be achievable in vivo.

The subject of non-CB_1_, non-CB_2_ targets has been comprehensively reviewed recently [92,96]. The Stella review introduces the novel AI receptors for biologically active indole compounds and describes their signal transduction via a Gs-mediated increase in cAMP production [92]. The Reggio review discusses opportunities for overlap in agonist activity among phylogenetically closely related GPCRs, as well as the potential for modifications in pharmacodynamic outcomes based upon heterodimerization or clustering interactions with other GPCRs [96]. The data reviewed herein argue for alternative mechanisms for cannabimimetic indoles to act via orphan or documented GPCRs, or non-GPCR mechanisms, by which AAI analogs could influence behaviors beyond their demonstrated agonist activity at the CB_1_ receptors. 

## 5. The Ultimate Diversion of Cannabimimetic Indoles: Spice/K2

For thousands of years, people have experimented with and intentionally consumed or administered novel chemical substances, experienced or observed and recorded their pharmacological effects, and speculated on their mechanisms of action. Preparations of chemicals that produced central nervous system effects such as euphoria, intoxication, stimulation, hallucinations, numbness, analgesia, and anesthesia were often adopted in medical, religious, and recreational practices. Records of preparation methods and pharmacological effects date back to the dawn of writing. With the advent of scientific methods and the disciplines of pharmacology and medicinal chemistry in the nineteenth century, medicinal chemistry data have been preserved in textbooks, scientific literature, patents, and a variety of other archival forms and are often freely available for reference on the internet. The scientific literature and online archives abound with research studies involving new synthetic cannabimimetics being synthesized and tested in in vitro and in vivo experiments, including numerous publications and forensic reports emphasizing the adverse consequences and potential for harm in humans that can be observed with exposure to extremely potent and efficacious synthetic cannabimimetic analogs. 

**Figure 7:**
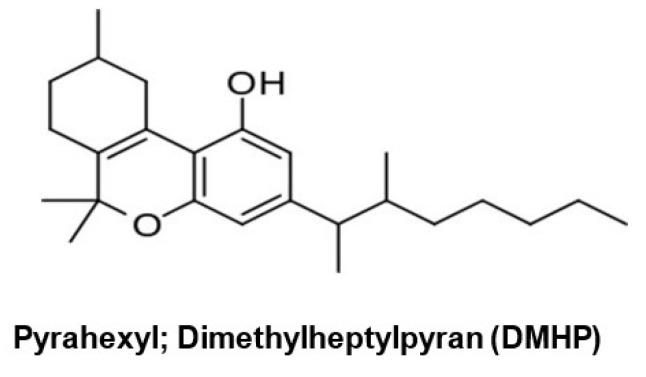
Structure of pyrahexyl (DMHP).

For example, Roger Adam’s and colleagues reported their testing of synthetic THC analogs in the 1940s [97,98,99], including a 1-2-dimethylheptyl analog of Δ^6a–10a^-tetrahydrocannabinol called pyrahexyl (Figure 7), which was several hundred-fold more potent than the pentyl analog. The potent activity observed after administration (oral consumption) of pyrahexyl did not go unconfirmed by the research scientists or unnoticed by the US Army [100], which included this compound in a development program for incapacitating chemical weapons [101]. The aim of this program was to develop compounds endowed with a “couch lock” or cataleptic effect, that is, non-lethal agents that could be used to incapacitate soldiers. For this reason, pyrahexyl, renamed dimethyl heptylpyran (DMHP) and assigned code number EA-2233 as the mixture of its eight stereoisomers, was included in chemical weapons research that proceeded from 1948 to 1975 at the Edgewood Arsenal in Maryland. In a remarkable effort of resolution and asymmetric synthesis, all eight stereoisomers of DMHP were synthesized, given individual codes EA-2233-1 through EA-2233-8, and investigated for bioactivity. EA-2233-2 was the most potent isomer and could induce confusion, sedation, and hallucinogenic effects at a dosage of 0.5–2.8 μg/kg, corresponding to 35–200 µg for a 70 kg adult. In general, an oral dosage of EA-2233 of 1–2 mg was sufficient to make all human subjects incapable of performing coordinated activities, such as those requested for military action, for as long as 2–3 days. Pyrahexyl was relatively safe, with a therapeutic index of 2000 in laboratory animals, but could occasionally induce severe hypotensive crises, hypothermia, and death, and was not eventually weaponized, in part due to the discovery of more efficacious and safer anticholinergic agents from the quinuclidinyl benzilate series, such as 3-quinuclidyl benzylate) [102].

**Figure 8:**
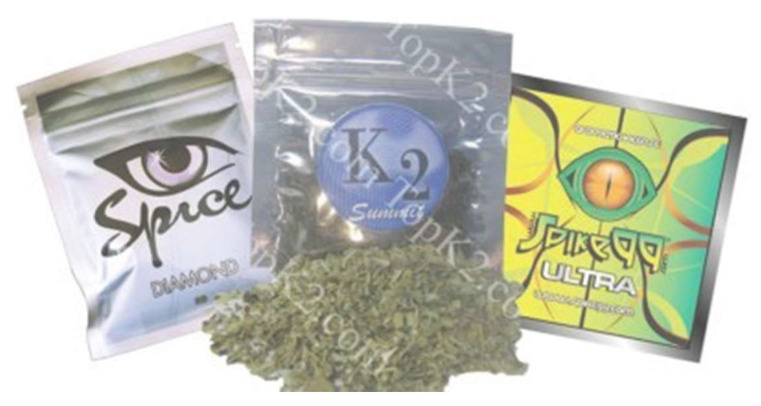
Examples of synthetic cannabimimetic-containing herbal formulations and packaging.

Structure–activity relationships of thousands of opiates and opioids, cannabinoids and synthetic cannabimimetics, dissociative anesthetics, steroids, stimulants, hallucinogens, sedative-hypnotics, and other psychoactive substances of potential abuse and dependence liability, many with synthetic methods and patents published, are readily accessible online to the scientific community and the public. Unfortunately, this information is also readily available to clandestine chemists who surreptitiously adopt or extend standard synthetic methods to manufacture and distribute illicit preparations of known psychoactive substances and to develop novel ones to sell on the illicit market as “designer drugs.” Based on information available on the internet and in scientific literature published by a wide variety of laboratories and research investigators, potent alkyl indole synthetic cannabimimetic chemicals began to be synthesized in bulk in the early 2000s and were often dissolved in a volatile solvent and sprayed on herbal products that were packaged and made widely available for purchase as “incense” or “spice” and subsequently smoked for their marijuana-like intoxicating properties (Figure 8). 

It was during this time that Jenny L. Wiley, a Distinguished Fellow at RTI International with a long history of pharmacological testing of cannabimimetics in laboratory animals, began encountering these illicit herbal products widely available for purchase in convenience stores and gas stations in Virginia and North Carolina. Since they were inappropriately labeled, she and Brian Thomas, the Senior Director of Analytical Chemistry and Pharmaceutics at RTI International, agreed to work together to assist the National Institute on Justice/US Drug Enforcement Agency (DEA) and the National Institute on Drug Abuse (NIDA) in the detection and identification of the synthetic cannabimimetics in these illicit drug products and the characterization of their in vitro cannabinoid receptor affinity and efficacy and in vivo behavioral effects in laboratory animal models of cannabimimetic activity. The results of these investigations, when published in peer-reviewed literature, were intended to facilitate regulation and enforcement, as well as the development of therapeutic treatments for adverse effects, overdose, and substance use disorders.

The spread of bulk synthetic cannabimimetics and synthetic cannabimimetic-containing herbal “spice” blends across international borders occurred rapidly, with products containing JWH-018 accounting for 76% of the 2423 herbal products seized, tested, and reported to the US DEA through the National Forensic Laboratory Information System (NFLIS) in 2010. Even though they were clearly capable of and used to produce profound intoxication, these products were often labeled “not for human consumption” and marketed as “herbal incense” or other misnomers to avoid prosecution by the DEA under the Federal Analogue Act. Unfortunately, the increased availability and use of these potent and efficacious cannabimimetic-containing products led to extreme intoxication, incapacitation, and an increasing number of calls to US Poison Control Centers, which prompted the DEA in March 2011 to use its emergency scheduling authority to temporarily place five of the most commonly encountered synthetic cannabimimetics into the Controlled Substances Act (CSA) as Schedule I; specifically: 1-pentyl-3-(1- naphthoyl)indole (JWH-018), 1-butyl-3-(1-naphthoyl)indole (JWH-073), 1-[2-(4-morpholinyl)ethyl]-3-(1-naphthoyl)indole (JWH-200), 5-(1,1-dimethylheptyl)-2-[(1R,3S)-3-hydroxycyclohexyl]-phenol (CP-47,497), and 5-(1,1-dimethyloctyl)-2-[(1R,3S)-3-hydroxycyclohexyl]-phenol (cannabicyclohexanol; CP-47,497 C8 homolog). This action was deemed necessary by the Administrator of the DEA to avoid an imminent hazard to public safety. As a result, the full effect of the CSA and its implementing regulations, including criminal, civil, and administrative penalties, sanctions, and regulatory controls of Schedule I substances, was brought to bear against the manufacture, distribution, possession, importation, and exportation of these substances and their herbal formulations. The percentage of illicit products containing these five agents seized or otherwise encountered and reported to the DEA decreased from 76% in 2010 to 20% in 2011. However, *a second generation of “legal” synthetic cannabimimetics was already being manufactured and distributed to replace the banned ones, such that during the same timeframe, 2010–2011, the total number of seizures and encounters of illicit products containing positively identified synthetic cannabimimetics increased 10-fold, to over 22,000.* In March of 2012, the DEA used its authority to extend the temporary placement of the five banned agents in Schedule 1 by 6 months. In July of 2012, the FDA Safety and Innovation Act (FDASIA) was passed. It included the Synthetic Drug Abuse Prevention Act that placed several more synthetic cannabimimetic analogs [1-hexyl-3-(1-naphthoyl)indole (JWH-019); 1-pentyl-3-(2-methoxyphenylacetyl)indole (JWH-250); 1-pentyl-3-[1-(4-methoxynaphthoyl)]indole (JWH-081); 1-pentyl-3-(4-methyl-1-naphthoyl)indole (JWH-122); 1-pentyl-3-(4-chloro-1-naphthoyl)indole (JWH-398); 1-(5-fluoropentyl)-3-(1-naphthoyl)indole (AM2201); 1-(5-fluoropentyl)-3-(2-iodobenzoyl)indole (AM694); 1-pentyl-3-[(4-methoxy)-benzoyl]indole (SR-19 and RCS-4); 1-cyclohexylethyl-3-(2-methoxyphenylacetyl)indole (SR-18 and RCS-8); 1-pentyl-3-(2-chlorophenylacetyl)indole (JWH-203), as well as specific synthetic stimulants and hallucinogens, under Schedule 1. It also increased the time that a substance remains in emergency Schedule I status from 1 year to 2, and increased the possible extension period from 6 months to 1 year. 

The DEA exercised its emergency scheduling authority again in 2013, 2014, 2015, 2016, 2017, 2019, and in 2021, as it continued to add new cannabimimetic substances under Schedule 1 of the CSA. For example, in 2013, three additional synthetic cannabimimetic analogs [1-pentyl-1H-indol-3-yl)(2,2,3,3-tetramethylcyclopropyl)methanone (UR-144); [1-(5-fluoro-pentyl)-1H-indol-3-yl](2,2,3,3-tetramethylcyclopropyl)methanone (5-fluoro-UR-144, XLR11), and N-(1-adamantyl)-1-pentyl-1H-indazole-3-carboxamide (APINACA, AKB48) were placed under schedule 1 of the CSA. In 2014, the synthetic cannabimimetics quinolin-8-yl 1-pentyl-1H-indole-3-carboxylate (PB-22; QUPIC); quinolin-8-yl 1-(5-fluoropentyl)-1H-indole-3-carboxylate (5-fluoro-PB-22; 5F-PB-22); N-(1-amino-3-methyl-1-oxobutan-2-yl)-1-(4-fluorobenzyl)-1H-indazole-3-carboxamide (AB-FUBINACA); and N-(1-amino-3,3-dimethyl-1-oxobutan-2-yl)-1-pentyl-1H-indazole-3-carboxamide (ADB-PINACA) were added. In 2015, the DEA included the synthetic cannabimimetics N-(1-amino -3-methyl-1-oxobutan-2-yl)-1-(cyclohexylmethyl)-1H-indazole-3-carboxamide (AB-CHMINACA); N-(1-amino-3-methyl-1-oxobutan-2-yl)-1-pentyl-1H-indazole-3-carboxamide (AB-PINACA); [1-(5-fluoropentyl)-1H-indazol-3-yl](naphthalen-1-yl)methanone (THJ-2201), and in 2016 added N-(1-amino-3,3-dimethyl-1-oxobutan-2-yl)-1-(cyclohexylmethyl)-1H-indazole-3-carboxamide (common names MAB-CHMINACA and ADB-CHMINACA), to the rapidly expanding list of Schedule 1 substances. Another DEA scheduling order was published in 2017 for six more synthetic cannabimimetic analogs appearing in illicit products: methyl 2-(1-(5-fluoropentyl)-1H-indazole-3-carboxamido)-3,3-dimethylbutanoate [5F-ADB; 5F-MDMB-PINACA]; methyl 2-(1-(5-fluoropentyl)-1H-indazole-3-carboxamido)-3-methylbutanoate [5F-AMB]; N-(adamantan-1-yl)-1-(5-fluoropentyl)-1H-indazole-3-carboxamide [5F-APINACA, 5F-AKB48]; N-(1-amino-3,3-dimethyl-1-oxobutan-2-yl)-1-(4-fluorobenzyl)-1H-indazole-3-carboxamide [ADB-FUBINACA]; methyl 2-(1-(cyclohexylmethyl)-1H-indole-3-carboxamido)-3,3-dimethylbutanoate [MDMB-CHMICA, MMB-CHMINACA]; and methyl 2-(1-(4-fluorobenzyl)-1H-indazole-3-carboxamido)-3,3-dimethylbutanoate [MDMB-FUBINACA], including their optical, positional, and geometric isomers, salts, and salts of isomers under schedule I. In 2019, ethyl 2-(1-(5-fluoropentyl)-1H-indazole-3-carboxamido)-3,3-dimethylbutanoate (5F-EDMB-PINACA); methyl 2-(1-(5-fluoropentyl)-1H-indole-3-carboxamido)-3,3-dimethylbutanoate (5F-MDMB-PICA); *N*-(adamantan-1-yl)-1-(4-fluorobenzyl)-1H-indazole-3-carboxamide (common names include FUB-AKB48; FUB-APINACA; AKB48 N-(4-fluorobenzyl)); 1-(5-fluoropentyl)-*N*-(2-phenylpropan-2-yl)-1H-indazole-3-carboxamide (common names of 5F-CUMYL-PINACA; SGT-25); and (1-(4-fluorobenzyl)-1H-indol-3-yl)(2,2,3,3-tetramethylcyclopropyl) methanone (FUB-144), and their optical, positional, and geometric isomers, salts, and salts of isomers were placed under schedule I; with the addition of these analogs made permanent in March of 2021. Effective as of June, 2021, the DEA has also included naphthalen-1-yl 1-(5-fluoropentyl)-1H-indole-3-carboxylate (NM2201 or CBL2201); N-(1-amino-3-methyl-1-oxobutan-2-yl)-1-(5-fluoropentyl)-1H-indazole-3-carboxamide (5F-AB-PINACA); 1-(4-cyanobutyl)-N-(2-phenylpropan-2-yl)-1H-indazole-3-carboxamide (other names: 4-CN-CUMYL-BUTINACA, 4-cyano-CUMYL-BUTINACA; 4-CN-CUMYL BINACA, CUMYL-4CN-BINACA, or SGT-78); methyl 2-(1-(cyclohexylmethyl)-1H-indole-3-carboxamido)-3-methylbutanoate (MMB-CHMICA or AMB-CHMICA); and 1-(5-fluoropentyl)-N-(2-phenylpropan-2-yl)-1H-pyrrolo[2,3-b]pyridine-3-carboxamide (5F-CUMYL-P7AICA) under Schedule 1 on a permanent basis. 

**Figure 9:**
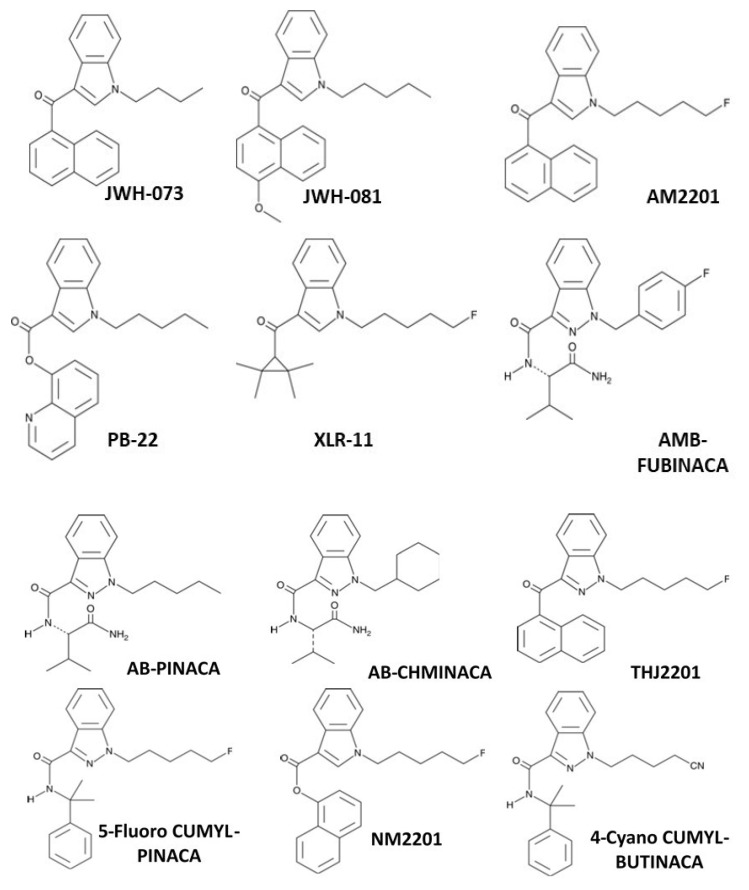
Chemical structures/IUPAC names of selected Schedule I synthetic cannabimimetics.

Presently, well over 40 novel synthetic cannabimimetic chemicals have been defined as Schedule 1 controlled substances by the DEA to discourage their further manufacture, distribution, and use (e.g., see Figure 9). However, the illicit drug market persists as new compounds are immediately created to evade detection, regulation, and law enforcement. This iterative cycle of synthesis, use, detection, identification, and banning of chemical substances has had the undesired effect of increasing the chemical diversity of illicit analogs being distributed in these products, thereby exposing users to a wider variety of compounds of unknown pharmacological activity and potential long-term negative consequence, while having a limited positive effect on the aggregate distribution and use. 

Over the last few decades, we have witnessed a growing commodification of psychoactive substances, including a diverse range of new chemical entities not controlled under drug laws. During a time of increasing legalization and use of medicinal and recreational cannabis and cannabinoid concentrates, a concurrent drug phenomenon has become largely defined by both the growing number of novel synthetic chemicals being detected from increasingly broad chemical and pharmacological families and the open sale of many of these substances as ‘legal highs’, ‘bath salts’, or ‘research chemicals’ in commercial venues and online shops, as well as by individual street-level drug dealers [103,104]. Over 400 new psychoactive substances were detected in Europe’s drug market in 2019, with extremely potent synthetic cannabimimetics, cathinones, arylcyclohexylamines, and opioids being the most prevalent classes of compounds posing significant health and social impact concerns. Reports of cannabis adulterated with new synthetic cannabimimetics, such as MDMB-4en-PINACA, being sold to unsuspecting recreational or medicinal cannabis users highlight the new and potentially growing risks of the inadvertent consumption of these illicit and relatively unknown substances [105]. Thus, the vernacular of designer drugs and new drug substances has been refined and replaced over time with ‘new psychoactive substance’ (NPS), increasingly being used in the rapidly evolving regulatory framework encompassing the legally contentious concept of use and misuse of psychoactive substances in our society. 

The current scheduling of new psychoactive substances in the US includes the specific mention of a variety of compounds as *Schedule I cannabimimetic agents*, “unless specifically exempted or unless listed in another schedule”, including “any material, compound, mixture, or preparation which contains any quantity of cannabimimetic agents, or which contains their salts, isomers, and salts of isomers is possible within the specific chemical designation” (Synthetic Drug Abuse Prevention Act of 2012). This act also defines cannabimimetic agents more broadly in terms of elements of their chemical scaffold and their substituents that have been demonstrated to be important for cannabimimetic activity (i.e., pharmacophores)—“The term cannabimimetic agents means any substance that is a cannabinoid receptor type 1 (CB_1_ receptor) agonist as demonstrated by binding studies and functional assays within any of the following structural classes:

- 2-(3-hydroxycyclohexyl)phenol with substitution at the 5-position of the phenolic ring by alkyl or alkenyl, whether or not substituted on the cyclohexyl ring to any extent.
- 3-(1-naphthoyl)indole or 3-(1-naphthylmethane)indole by substitution at the nitrogen atom of the indole ring, whether or not further substituted on the indole ring to any extent, whether or not substituted on the naphthoyl or naphthyl ring to any extent.
- 3-(1-naphthoyl)pyrrole by substitution at the nitrogen atom of the pyrrole ring, whether or not further substituted in the pyrrole ring to any extent, whether or not substituted on the naphthoyl ring to any extent.
- 1-(1-naphthylmethylene)indene by substitution of the 3-position of the indene ring, whether or not further substituted in the indene ring to any extent, whether or not substituted on the naphthyl ring to any extent.
- 3-phenylacetylindole or 3-benzoylindole by substitution at the nitrogen atom of the indole ring, whether or not further substituted in the indole ring to any extent, whether or not substituted on the phenyl ring to any extent.”

Unfortunately, broad definitions of core structural components may include compounds that have structural similarity to cannabimimetic agents but do not produce cannabimimetic effects. In addition, the inclusion of cannabinoid receptor binding studies and functional assay data as criteria for declaration of a cannabimimetic agent is problematic because these experiments can be complex, must be performed properly by a qualified laboratory with appropriate controls, and the results and conclusions carefully reviewed and confirmed prior to use in a court of law. Finally, the identity of the chemical constituents in the products are often identified, characterized, and banned, but these chemicals may differ dramatically from the chemical exposures that are produced during the use of these compounds, either due to degradation, thermolysis, or rapid metabolic conversion. 

When synthetic cannabimimetics are encountered in bulk, the “pure” compounds are commonly in the form of fine crystalline powders but may also be amorphous solids, with colors ranging across white, grey, brown, and yellow hues. The quality of these synthetic chemicals often fails to meet pharmaceutical standards for purity or identification and labeling of all active ingredients, excipients, or impurities exceeding an acceptable standard percentage or estimated daily dose exposure [106,107,108,109]. In addition, most of the chemical ingredients are improperly identified on customs declarations, using a variety of inaccurate chemical descriptors or inappropriate descriptions (e.g., herbal incense). The purity of these synthetic preparations varies widely and appears to be poorly controlled. In some instances, seized bulk synthetic cannabimimetic chemicals have been found to be contaminated with a variety of synthetic by-products and intermediates originating from the synthetic procedures employed, and a variety of structural analogs have been shown to degrade at commonly encountered room temperature exposures [110]. The proper handling, storage, separation, and detection of these novel chemicals in complex matrix and elucidation of the exact chemical structure often requires the use of several sophisticated analytical instruments and laboratory techniques and the interpretation of complex datasets that together can provide sufficient integrated molecular information to confirm identity. Moreover, the analytical methods used for legal or forensic purposes must also be validated and shown to provide suitably accurate, specific, and reliable information, which adds to the cost and complexity involved in either targeted or broad-spectrum methods [111,112]. Finally, in vitro and in vivo laboratory studies are increasingly used to provide evidence that novel chemicals that are being encountered on the illicit market are cannabimimetics that bind to and activate CB_1_ cannabinoid receptors [113,114,115].

The evolution of synthetic cannabimimetics has involved modification of both chemical scaffolds and substituents that extend beyond literature precedent or established cannabinoid receptor binding affinity/efficacy studies [116,117] and have tended to produce novel chemicals whose volatility and thermal stability are compromised as compared to JWH-018 [118,119]. Thermolysis and the formation of degradation products of synthetic cannabimimetic chemicals is a function of their chemical structure and high temperature exposure, such as during vaporization or combustion processes employed for inhalation. For example, halogenation of synthetic cannabimimetic analogs has been widely used to evade detection and circumvent law enforcement actions; however, this modification leads to increased thermal lability, specifically, thermolytically induced dehalogenation and desaturation of the alkyl side chain [120,121]. In other instances, synthetic analogs such as UR-144 and XLR-11 containing a sterically strained ring system in lieu of the alkyl sidechain have been shown to rapidly decompose to ring-opened and/or dehalogenated species [110,122,123,124]. Carboxamide synthetic analogs, including the PICA and FUBINACA analogs associated with fatalities and so-called “zombie outbreaks”, have also been shown to undergo rapid thermolytic degradation under elevated temperature exposures that may be relevant to combustion or vaporization and inhalation routes of administration [125]. Even relatively modest changes in chemical structure can have a profound influence on volatility and thermal stability and pharmacokinetics and pharmacodynamics, leading to dramatic differences in chemical exposure due to thermal degradation and transfer of chemicals into the gas vapor phase during heating or combustion and inhalation, or differences in their adsorption, distribution, metabolism, elimination and pharmacodynamic impact over time [126,127]. 

The variability in the thermal stability of synthetic cannabimimetic analogs appears to span the entire range, from compounds that volatilize intact when heated, with little to no thermal degradation, to compounds that degrade slowly at room temperature and entirely decompose during heating before they can produce a vapor containing the parent compound for inhalation. Thus, when smoking vessels such as pipes or other devices which have been used to combust or vaporize and inhale synthetic cannabimimetic-containing herbal blends are examined for residual chemicals, parent compound(s) may be absent and replaced by degradants and thermolysis products. For example, individuals have been reported to primarily excrete metabolites of the thermal degradants of synthetic cannabimimetics formed during combustion and inhalation of herbal formulations, as opposed to excreting metabolites of the intact drug substance detected on the plant material (i.e., the primary exposure during combustion and inhalation is to the thermal degradant). In this case, the detection of mono-hydroxylated metabolites of UR-144 (LC-MS-MS) and mono-hydroxylated/with hydration metabolites of the UR-144 pyrolysis product (GC-MS) was found to be the most useful method of establishing UR-144 ingestion [128]. Unfortunately, the thermal degradants that are formed during the heating of synthetic cannabimimetics often include compounds with known toxicity. For example, when incrementally heated at 200, 400, 600, and 800 °C, the alkyl indole NNEI decomposes to form a variety of compounds, including naphthylamine (a carcinogen) and pentylindole, whereas the structurally analogous indazole MN-18 appears to volatilize with significantly less thermolysis. However, many of the carboxamide-containing synthetic cannabimimetics also appear to be susceptible to decomposition and liberation of hydrogen cyanide when heated rapidly to 800 °C, which was confirmed and quantified via LC-MS/MS [127]. These results suggest that the liberation of toxic degradants, including hydrogen cyanide released during the heating and inhalation of synthetic indazole carboxamide-type compounds, could have significant health impacts on human users of synthetic cannabimimetic containing herbal formulations [127]. 

**Figure 10:**
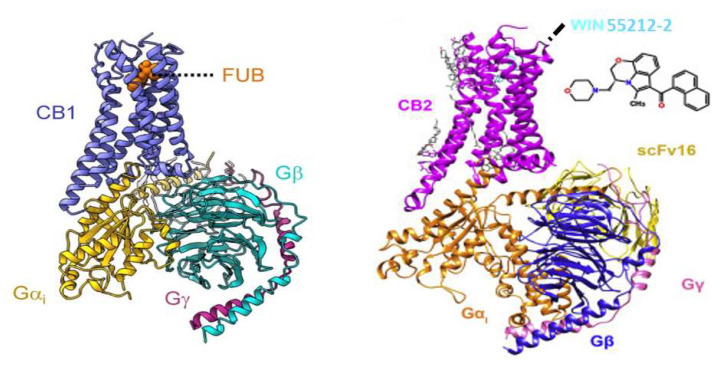
Cryo-electronmicrograph depicting structures of CB1 cannabinoid receptor stimulated by FUBINACA (FUB) and engaging Giα1β1γ2 (**left**). Image reprinted with permission from Cell 176, Krishna Kumar, K. et al., *Structure of a Signaling Cannabinoid Receptor 1 G Protein Complex*., copyright 2019, with permission from Elsevier [138]; and CB2 cannabinoid receptor stimulated by WIN55212-2 and engaging Giα1β1γ2 (**right**). Image reprinted with permission from Cell 180, Xing, C. et al., *Cryo-EM Structure of the Human Cannabinoid Receptor CB2-G_i_ Signaling Complex.*, copyright 2020, with permission from Elsevier [139].

There has been a continued increase in the diversity of both the synthetic cannabimimetic chemicals being manufactured and used and the variety of formulations being encountered in the illicit market, such as in vape pens and tinctures and edible products, in addition to herbal blends and bulk drug substances [105]. These illicit products continue to have no oversight ensuring the accuracy or validity of their label claims and provide little or no guidance on proper storage, indications for use or dose titration, or information on commonly encountered adverse side effects. Thus, the effects that are produced in consumers can vary considerably, and can occasionally be debilitating and lethal, produce dependence and withdrawal, and range dramatically in intensity and duration depending upon dose and route of administration (for example, see [129,130,131,132,133]). Nevertheless, these products remain of considerable interest to individuals who pursue intoxication while enabling their chemical use to remain undetected and clear of legal regulations and criminal consequences (e.g., individuals subjected to periodic urinalysis for employment or military/civil service [134,135]). The evolving supply chain of new chemical scaffolds in designer drugs challenges forensic laboratories and public health resources that rely upon rapid analysis of bulk drug substances, dosage formulations, and drugs and their metabolites in biological fluids to derive an appropriate legal response or treatment strategy. In response, drug-testing laboratories use increasingly sophisticated chemical analysis methods and bioanalytical technologies, which also challenge the clinicians, analytical chemists, and authorities who must properly interpret the complex analytical results and implement appropriate medical or regulatory responses. Even though a chemical prototype may have a long history of use and considerable literature, each new chemical entity is essentially a pharmacological unknown with the inherent potential to produce unanticipated effects in users or their descendants [117]. For example, G-protein promiscuity and signaling bias has been shown to be an important pharmacological property that may differentiate between phytocannabinoids and synthetic cannabimimetics and their relative ability to produce tolerance and dependence and other pharmacological effects [136,137]. Synthetic alkyl indole compounds are able to activate CB_1_ and CB_2_ cannabinoid receptors, and the selectivity can make a difference in outcomes of G protein signaling (see Figure 10).

Because of the technical difficulty in the detection and characterization of new designer drugs of abuse, estimates of their extent of use and effects produced must be derived using survey data, Poison Control Center data, and many other resources to produce accurate estimates. Thus, there is a significant need for a comprehensive discussion on synthetic cannabimimetic designer drugs that recognizes their potential threat to society, presents the ongoing challenges confronting the various approaches to detection and identification, and informs the development of improved solutions for use in legislation, law enforcement, harm reduction, and clinical treatment. 

## 6. Conclusions: Scientists and Entrepreneurs: Who Takes Social Responsibility?

Researchers in the field of cannabinoid biochemistry, physiology, and pharmacology are conscious of research ethics in developing hypotheses and conducting investigations. Now, we are entering into an era in which consumers are expected to make judgments without having the advantage of education in chemical and biological sciences or training in the scientific method of applying research observations to developing and testing hypotheses, analyzing data, and drawing conclusions. Researchers come to conclusions that are directed at understanding mechanisms and discerning fundamental differences between physiology versus pathophysiology of the disease. Consumers are expected to make conclusions about whether plant products and their derivatives are useful for their health and safe for their use. Entrepreneurs and business developers make conclusions based upon their goal to commercialize cannabinoid plant products and compounds derived therefrom. 

The diverse goals for the application of current knowledge are dependent upon stakeholders who are motivated to provide funding support. For scientific researchers, funding support is obtained competitively from governmental sources derived from public tax dollars or foundations directed at curing diseases with public donations. Researchers are accountable to demonstrate that the work will be peer-reviewed and made publically available so that other scientists can build upon the work. Indeed, it is generally expected that the researcher has a history of publishing work before grant proposals are funded. 

If funding comes from private sources (i.e., the pharmaceutical industry), researchers are generally expected to keep their work confidential to protect intellectual property. Nearly a dozen pharmaceutical companies contributed to developing and characterizing AAI compounds described in this review, each espousing the goal to provide consumers with medicines that are effective and safe. Impediments to ultimately marketing a medicine may occur at any of the steps in the drug development process. This is a risk that a legitimate pharmaceutical company is willing to take if it wishes to maintain its reputation for providing safe and effective medicines. Many projects are terminated based upon poor preclinical responses in models, failed clinical outcomes, untoward side effects, or adverse events. It is interesting to note that the preclinical research reviewed herein has not resulted in a marketed medicine. 

When the properties of JWH-018 were published, researchers did not anticipate the abuse and misuse of the compound. In hindsight, one can imagine entrepreneurs discussing whether or not the compound could be used to become high. Would it circumvent drug laws that existed at the time? How might it be distributed? There are potential marketing strategies that a less-than-ethical commercial enterprise might consider in its effort to gain profit from the application of available research methods. Entrepreneurs who marketed unscheduled AAI and other cannabimimetic compounds under the guise of “legal marijuana” bear much of the responsibility for the misuse of the compounds. They sold products without testing for safety. They sold an impure product. Even when packaging included a label to the contrary, one can surmise the intention was for users to smoke or ingest the product. While maintaining the “letter of the law” in assuring that compounds they sold were not listed by the US DEA as schedule 1, they bypassed the intent of the law. By indicating that compounds are not illegal, they led consumers to believe that the safety of these products had been tested and that these compounds could be used without harm.

What can scientists do to promote research and avoid public mis- or disinformation? As one government funding goal is to train the next generation of researchers to keep the nation’s healthcare capabilities strong, part of the job of scientists is to educate students. However, another part is to educate consumers whose interests are limited to whether a plant product or synthetic compound can treat their maladies and if they can expect “side effects”. Scientists need to use accurate wording in scientific communications and avoid terms that are imprecise and lead to generalizations and misunderstandings. An example is “synthetic cannabinoid”, which incorrectly includes indole compounds that are neither cannabinoid in structure nor analogs of natural phytocannabinoids. Scientists should communicate to the public at their level of understanding and interest and still take the opportunity to teach consumers about cell biology, physiology, or pathology as it applies to the mechanism of action of medicines. Researchers also need to communicate about the importance of research and the scientific method.

There are no good drugs or bad drugs; rather, there are good uses and bad uses for compounds whether found in nature or synthesized by design. The story of the AAI and analogs described in this review aptly demonstrates this pharmacological principle.

## Figures and Tables

**Table 1:** Cannabimetic indole analogs exhibiting improved CB2/CB1 receptor selectivity.

Name	N1	C2	C3	CB_2_ K_i_ (nM)	CB_2_/CB_1_ Selectivity Ratio	Reference
JWH-015	Propyl	Methyl	1-naphthoyl	13.8	27	[57]
JWH-046	Propyl	Methyl	7-methyl-1-naphthoyl	16.0	21	[58]
JWH-120	Propyl	H	4-methyl-1-naphthoyl	6.1	170	[57]
JWH-267	Pentyl	H	2-methoxy-1-naphthoyl	7.2	54	[59]
JWH-151	Propyl	Methyl	6-methoxy-1-naphthoyl	30.0	>300	[59]
