# Correction: Howlett et al. The Spicy Story of Cannabimimetic Indoles. *Molecules* 2021, *26*, 6190

**DOI:** 10.3390/molecules29153456

**Published:** 2024-07-24

**Authors:** Allyn C. Howlett, Brian F. Thomas, John W. Huffman

**Affiliations:** 1 Department of Physiology and Pharmacology, Wake Forest School of Medicine, Winston-Salem, NC 27157, USA; 2 Department of Analytical Sciences, The Cronos Group, Toronto, ON M5V 2H1, Canada; 3 Department of Chemistry, Clemson University, Clemson, SC 29634, USA

Missing Conflicts of Interest

In the original publication [1], the Conflicts of Interest statement of Brian F. Thomas was not included. The updated Conflicts of Interest should be as follows: Brian F. Thomas was employed by the company Cronos Group. The remaining authors declare no conflicts of interest. The Cronos Group had no role in the design of this review article; in the collection, analyses, or interpretation of data discussed herein; in the writing of the manuscript; or in the decision to publish the results. 

Due to the expiration of Brian F. Thomas’ previous email address, we have updated Brian F. Thomas’ email to brian.f.thomas01@gmail.com to facilitate reader contact.

The authors state that the scientific conclusions are unaffected. This correction was approved by the Academic Editor. The original publication has also been updated.
